# Prevalence of Dyslipidemia and Availability of Lipid-Lowering Medications Among Primary Health Care Settings in China

**DOI:** 10.1001/jamanetworkopen.2021.27573

**Published:** 2021-09-29

**Authors:** Yuan Lu, Haibo Zhang, Jiapeng Lu, Qinglan Ding, Xinyue Li, Xiaochen Wang, Daqi Sun, Lingyi Tan, Lin Mu, Jiamin Liu, Fang Feng, Hao Yang, Hongyu Zhao, Wade L. Schulz, Harlan M. Krumholz, Xiangbin Pan, Jing Li

**Affiliations:** 1 Center for Outcomes Research and Evaluation, Yale-New Haven Hospital, Section of Cardiovascular Medicine, Department of Internal Medicine, Yale School of Medicine, New Haven, Connecticut; 2 National Clinical Research Center for Cardiovascular Diseases, National Health Commission Key Laboratory of Clinical Research for Cardiovascular Medications, State Key Laboratory of Cardiovascular Disease, Fuwai Hospital, Chinese Academy of Medical Sciences and Peking Union Medical College, National Center for Cardiovascular Diseases, Beijing, China; 3 Department of Biostatistics, Yale University, New Haven, Connecticut; 4 Fuwai Hospital Chinese Academy of Medical Sciences, Shenzhen, China

## Abstract

**Importance:**

Dyslipidemia, the prevalence of which historically has been low in China, is emerging as the second leading yet often unaddressed factor associated with the risk of cardiovascular diseases. However, recent national data on the prevalence, treatment, and control of dyslipidemia are lacking.

**Objective:**

To assess the prevalence, treatment, and control of dyslipidemia in community residents and the availability of lipid-lowering medications in primary care institutions in China.

**Design, Setting, and Participants:**

This cross-sectional study used data from the China-PEACE (Patient-Centered Evaluative Assessment of Cardiac Events) Million Persons Project, which enrolled 2 660 666 community residents aged 35 to 75 years from all 31 provinces in China between December 2014 and May 2019, and the China-PEACE primary health care survey of 3041 primary care institutions. Data analysis was performed from June 2019 to March 2021.

**Exposures:**

Study period.

**Main Outcomes and Measures:**

The main outcome was the prevalence of dyslipidemia, which was defined as total cholesterol greater than or equal to 240 mg/dL, low-density lipoprotein cholesterol (LDL-C) greater than or equal to 160 mg/dL, high-density lipoprotein cholesterol (HDL-C) less than 40 mg/dL, triglycerides greater than or equal to 200 mg/dL, or self-reported use of lipid-lowering medications, in accordance with the 2016 Chinese Adult Dyslipidemia Prevention Guideline.

**Results:**

This study included 2 314 538 participants with lipid measurements (1 389 322 women [60.0%]; mean [SD] age, 55.8 [9.8] years). Among them, 781 865 participants (33.8%) had dyslipidemia. Of 71 785 participants (3.2%) who had established atherosclerotic cardiovascular disease (ASCVD) and were recommended by guidelines for lipid-lowering medications regardless of LDL-C levels, 10 120 (14.1%) were treated. The overall control rate of LDL-C (≤70 mg/dL) among adults with established ASCVD was 26.6% (19 087 participants), with the control rate being 44.8% (4535 participants) among those who were treated and 23.6% (14 552 participants) among those not treated. Of 236 579 participants (10.2%) with high risk of ASCVD, 101 474 (42.9%) achieved LDL-C less than or equal to 100 mg/dL. Among participants with established ASCVD, advanced age (age 65-75 years, odds ratio [OR], 0.63; 95% CI, 0.56-0.70), female sex (OR, 0.56; 95% CI, 0.53-0.58), lower income (reference category), smoking (OR, 0.89; 95% CI, 0.85-0.94), alcohol consumption (OR, 0.87; 95% CI, 0.83-0.92), and not having diabetes (reference category) were associated with lower control of LDL-C. Among participants with high risk of ASCVD, younger age (reference category) and female sex (OR, 0.58; 95% CI, 0.56-0.59) were associated with lower control of LDL-C. Of 3041 primary care institutions surveyed, 1512 (49.7%) stocked statin and 584 (19.2%) stocked nonstatin lipid-lowering drugs. Village clinics in rural areas had the lowest statin availability.

**Conclusions and Relevance:**

These findings suggest that dyslipidemia has become a major public health problem in China and is often inadequately treated and uncontrolled. Statins were available in less than one-half of the primary care institutions. Strategies aimed at detection, prevention, and treatment are needed.

## Key Points

**Question:**

What is the prevalence of dyslipidemia in Chinese adults and what are the treatment and control rates of elevated low-density lipoprotein cholesterol in both primary and secondary prevention populations?

**Findings:**

In this cross-sectional study of 2 314 538 community residents, 33.8% had dyslipidemia, 3.2% had established atherosclerotic cardiovascular disease (ASCVD), and 10.2% had high risk of ASCVD; 26.6% of those with ASCVD and 42.9% of those at high risk of ASCVD achieved low-density lipoprotein cholesterol control targets. Statins were available in 49.7% of the primary care institutions surveyed, with the lowest availability in rural village clinics.

**Meaning:**

These findings suggest that dyslipidemia has become a major public health problem in China and is often inadequately treated and uncontrolled.

## Teaser

This cross-sectional study assesses the prevalence, treatment, and control of dyslipidemia in community residents and the availability of lipid-lowering medications in primary care institutions in China.

## Introduction

China is in the midst of an epidemiological transition in which cardiovascular diseases (CVDs) have replaced infectious diseases as the leading cause of death.^1^ Currently, CVD in China accounts for more than 40% of the causes of death.^2^ There are concerns that dyslipidemia, the prevalence of which historically was low in China, is emerging as the second leading yet often unaddressed factor associated with the risk of CVD.^3^

Recent national studies^4,5^ have assessed the overall prevalence of dyslipidemia and achievement of low-density lipoprotein cholesterol (LDL-C) lowering targets in the Chinese population. However, they did not assess the treatment rate and control rate of elevated LDL-C in both primary and secondary prevention populations. In addition, data are lacking on the availability of lipid-lowering medications in primary care institutions, which play an important role in preventing and managing chronic diseases. Assessing the prevalence, treatment, and control patterns of dyslipidemia and associated characteristics in community residents can help identify subgroups of individuals who will be the target population for interventions to reduce cardiovascular risk. Moreover, assessing the availability of lipid-lowering medications in primary care institutions is critical for assisting the development of policies to mitigate the burden of dyslipidemia.

The China-PEACE (Patient-Centered Evaluative Assessment of Cardiac Events) Million Persons Project (MPP), a large-scale population-based screening project, is an ideal platform to study dyslipidemia in Chinese adults, given the large size of this data set and the recruitment of participants at the community level. Accordingly, in this cross-sectional study, we leveraged the China-PEACE MPP and a national survey of primary care institutions to describe the prevalence, treatment, and control of dyslipidemia and the availability of lipid-lowering medications in primary care institutions.

## Methods

### Participants and Study Design

The central ethics committee at the China National Center for Cardiovascular Disease and the institutional review board at Yale University approved this project. All enrolled participants provided written informed consent. This study follows the Strengthening the Reporting of Observational Studies in Epidemiology (STROBE) reporting guideline for cross-sectional studies.^6^

#### China-PEACE MPP

From December 2014 to May 2019, we used a purposive sampling method to select 189 sites (114 rural counties, 75 urban districts) across all 31 provinces in China.^7^ Sites were selected purposefully to reflect the diversity in geographical distribution, economic development, and population structure across the country (see details in eAppendix 1 in Supplement 1). At each site, participants were recruited by local staff via extensive publicity campaigns on television and in newspapers and were enrolled if they were aged 35 to 75 years and currently residents in the selected region. Participants were then screened for high risk of CVD using measurements of blood pressure, blood lipids, blood glucose, height, and weight; a questionnaire assessing cardiovascular-related health status was also administered. Of the 2 660 666 participants enrolled, 322 814 (12.1%) were excluded from this analysis because they lacked fasting blood lipid measurements or had implausible lipid values, and 23 314 (0.8%) were excluded because of missing data for other covariates (eFigure 1 in Supplement 1).

#### China-PEACE Primary Health Care Survey

From November 2016 to May 2017, we conducted a nationwide survey in 3529 primary care institutions in the China-PEACE MPP network.^8^ These institutions included 188 community health centers and 490 community health stations from the urban areas, and 286 township health centers and 2565 village clinics from the rural areas. In China, primary health care services are provided by community health centers and community health stations (1 level below) in urban areas and by township health centers and village clinics (1 level below) in rural areas. The distribution of sampled primary care institutions across rural and urban areas reflected the national ratio of rural to urban institutions.^9^

### Data Collection and Variables

At the initial screening of China-PEACE MPP, participants underwent a lipid blood test performed by a rapid lipid analyzer using whole blood samples (CardioChek PA Analyzer; Polymer Technology Systems). Participants were considered in a fasting state if their last meals were taken more than 8 hours before. Total cholesterol (TC), triglycerides (TG), and high-density lipoprotein cholesterol (HDL-C) were measured. LDL-C was calculated with the Friedewald equation after excluding participants with TG greater than 400 mg/dL (to convert TG to millimoles per liter, multiply by 0.0113).

Dyslipidemia was defined as TC greater than or equal to 240 mg/dL (to convert to millimoles per liter, multiply by 0.0259), LDL-C greater than or equal to 160 mg/dL (to convert to millimoles per liter, multiply by 0.0259), HDL-C less than 40 mg/dL (to convert to millimoles per liter, multiply by 0.0259), or TG greater than or equal to 200 mg/dL, or self-reported use of lipid-lowering medications, in accordance with the 2016 Chinese Adult Dyslipidemia Prevention Guideline.^10^ We used the same definition among secondary prevention population to be consistent with previous national studies in China.^4,5^ Participants were considered as being treated for dyslipidemia if they reported using lipid-lowering medication (Western medicines or traditional Chinese medication [TCM]) within the last 2 weeks. Control of LDL-C was defined on the basis of atherosclerotic cardiovascular disease (ASCVD) risk stratification in accordance with the Chinese guideline (see details in eAppendix 2 in Supplement 1).^10^ Specifically, participants were considered as achieving LDL-C control targets if they had established ASCVD (ie, coronary heart disease or stroke) and LDL-C less than or equal to 70 mg/dL, or if they had an estimated 10-year ASCVD risk of greater than or equal to 10% and LDL-C less than or equal to 100 mg/dL, or if they had an estimated 10-year ASCVD risk of less than 10% and LDL-C less than or equal to130 mg/dL.

Information on the participants’ sociodemographic characteristics, health behaviors, medical history, and cardiovascular risk factors was recorded during in-person interviews as described elsewhere.^7^ Height and weight were collected using standard protocols, and body mass index was calculated by dividing the weight in kilograms by the square of height in meters. Obesity was defined as a body mass index of at least 28, in accordance with the recommendations of the Working Group on Obesity in China.^11^

The availability of lipid-lowering medications, including statins, nonstatins, and TCMs (see a complete list in eAppendix 3 in Supplement 1), was obtained from each primary care institution participating in the China-PEACE primary health care survey. We collected lists of medications in stock at the time of the survey from each participating primary care institution. For each medication on the list, we collected its generic name, brand name, and dosage.^8^

### Statistical Analysis

We described the prevalence of dyslipidemia in the overall study population and stratified by ASCVD risk. Because the indication of lipid-lowering therapy is based on ASCVD risk in current clinical guidelines,^10^ we described the treatment and control rates of LDL-C among participants with established ASCVD and high risk of ASCVD, respectively. Both groups were recommended by clinical guidelines to achieve LDL-C control targets to lower the risk of ASCVD. In a sensitivity analysis, we determined the age-standardized prevalence of dyslipidemia by adjusting observation weights to match the age and sex distributions in the 2010 Chinese Census.^12^

We developed multivariable mixed models with a logit link function and township-specific random intercepts (to account for geographical autocorrelation) to identify individual characteristics associated with the prevalence of dyslipidemia among all study participants and the control of LDL-C among participants with established and high risk of ASCVD, respectively. Finally, we assessed the availability of lipid-lowering medications in primary care institutions by calculating the proportion of participating institutions with a specific type of lipid-lowering medication in stock. We examined the availability by type of primary care institutions and region.

All analyses were conducted with R statistical software version 3.33 (R Project for Statistical Computing). All statistical testing was 2-sided, at a significance level of *P* < .05. Data analysis was performed from June 2019 to March 2021.

## Results

### Prevalence of Dyslipidemia Overall and in Subtypes

Our final sample of China-PEACE MPP included 2 314 538 participants (1 389 322 women [60.0%]; mean [SD] age, 55.8 [9.8] years) (eFigure 1 in Supplement 1), 1 369 160 of whom (59.2%) were from rural areas (Table 1). Overall, 781 865 participants (33.8%) had dyslipidemia, 71 785 (3.2%) had experienced prior cardiovascular events, and 236 579 (10.2%) had high risk of ASCVD.

**Table 1.** Characteristics of Participants in China Patient-Centered Evaluative Assessment of Cardiac Events Million Persons Project

Characteristics	Participants, No. (%)		
	Overall	With dyslipidemia	Without dyslipidemia
Total	2 314 538 (100.0)	781 865 (33.8)	1 532 673 (66.2)
Age, y			
35-44	346 630 (15.0)	104 166 (13.3)	242 464 (15.8)
45-54	729 548 (31.5)	243 426 (31.1)	486 122 (31.7)
55-64	727 975 (31.5)	260 271 (33.3)	467 704 (30.5)
65-75	510 385 (22.1)	174 002 (22.3)	336 383 (21.9)
Sex			
Male	925 216 (40.0)	349 896 (44.8)	575 320 (37.5)
Female	1 389 322 (60.0)	431 969 (55.2)	957 353 (62.5)
Urbanity			
Urban	945 378 (40.8)	340 014 (43.5)	605 364 (39.5)
Rural	1 369 160 (59.2)	441 851 (56.5)	927 309 (60.5)
Region			
Eastern	872 317 (37.7)	303 185 (38.8)	569 132 (37.1)
Western	783 088 (33.8)	262 460 (33.6)	520 628 (34.0)
Central	659 133 (28.5)	216 220 (27.7)	442 913 (28.9)
Education			
Primary school or lower	976 520 (42.2)	307 353 (39.3)	669 167 (43.7)
Middle school	755 316 (32.6)	257 997 (33.0)	497 319 (32.4)
High school	368 971 (15.9)	136 236 (17.4)	232 735 (15.2)
College or above	182 218 (7.9)	69 669 (8.9)	112 549 (7.3)
Unknown^a^	31 513 (1.4)	10 610 (1.4)	20 903 (1.4)
Annual household income, yuan^b^			
<10 000	447 743 (19.3)	140 275 (17.9)	307 468 (20.1)
10 000-50 000	1 275 123 (55.1)	427 161 (54.6)	847 962 (55.3)
>50 000	383 064 (16.6)	144 180 (18.4)	238 884 (15.6)
Unknown^a^	208 608 (9.0)	70 249 (9.0)	138 359 (9.0)
Marital status			
Married	2 151 920 (93.0)	726 565 (92.9)	1 425 355 (93.0)
Widowed, separated, divorced, single	135 022 (5.8)	46 019 (5.9)	89 003 (5.8)
Unknown^a^	27 596 (1.2)	9281 (1.2)	18 315 (1.2)
Health insurance status			
Insured	2 262 681 (97.8)	764 283 (97.8)	1 498 398 (97.8)
Uninsured	14 978 (0.6)	5261 (0.7)	9717 (0.6)
Unknown^a^	36 879 (1.6)	12 321 (1.6)	24 558 (1.6)
Medical history			
Myocardial infarction	16 920 (0.7)	8717 (1.1)	8203 (0.5)
Stroke	56 920 (2.5)	26 545 (3.4)	30 375 (2.0)
Cardiovascular disease risk factor			
Diabetes	150 433 (6.5)	74 262 (9.5)	76 171 (5.0)
Current smoker	443 055 (19.1)	171 938 (22.0)	271 117 (17.7)
Current drinker	544 902 (23.5)	197 074 (25.2)	347 828 (22.7)
Obesity^c^	362 392 (15.7)	170 600 (21.8)	191 792 (12.5)

^a^ Unknown reflects that the participants either refused to answer the question or did not know the answer.

^b^ The average conversion rate in 2019 was 6.91 yuan to $1.00 US.

^c^ Obesity is defined as a body mass index (weight in kilograms divided by height in meters squared) greater than or equal to 28.

**Figure 1.**
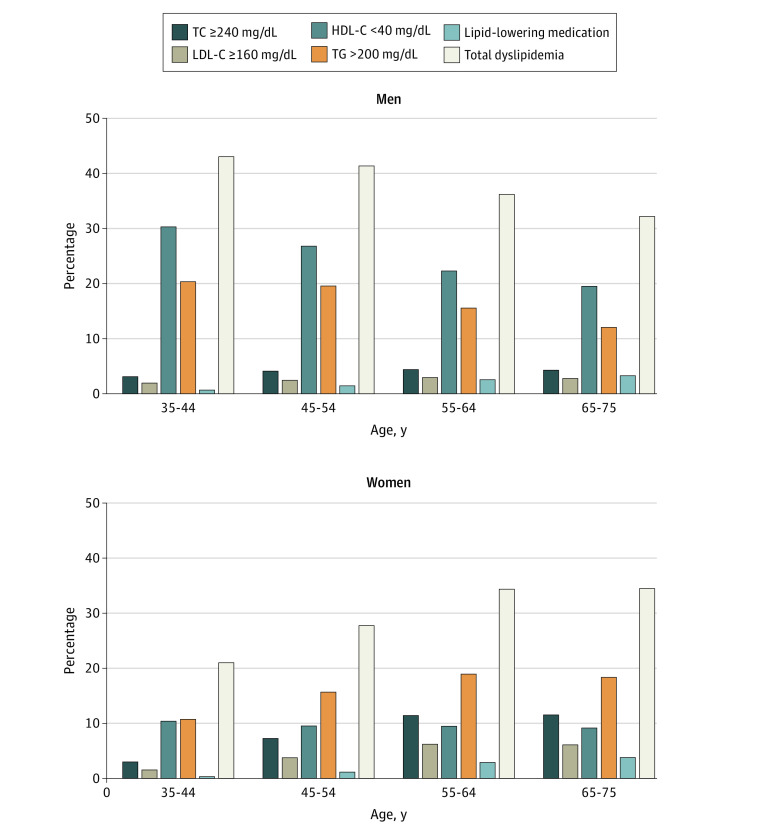
Prevalence of Abnormal Lipid Profiles Among China Patient-Centered Evaluative Assessment of Cardiac Events Million Persons Project Participants, by Age and Sex HDL-C indicates high-density lipoprotein cholesterol (to convert to millimoles per liter, multiply by 0.0259); LDL-C, low-density lipoprotein cholesterol (to convert to millimoles per liter, multiply by 0.0259); TC, total cholesterol (to convert to millimoles per liter, multiply by 0.0259); TG, triglycerides (to convert to millimoles per liter, multiply by 0.0113).

The prevalence of high TC was 7.1% (164 201 participants), that of high LDL-C was 4.0% (91 683 participants), that of high TG was 16.9% (390 244 participants), and that of low HDL-C was 15.6% (361 395 participants). Low HDL-C and high TG levels were the most common lipid abnormalities (46.2% [361 395 participants] and 49.9% [390 244 participants] of individuals with dyslipidemia, respectively), with mean (SD) HDL-C of 55.9 (15.9) mg/dL and mean (SD) TG of 139.9 (69.3) mg/dL (eFigure 2 in Supplement 1 and Figure 1). Using the 2010 Chinese Census data, we reported the age- and sex-standardized rate of overall dyslipidemia to be 34.1%.

In multivariable regression analysis, we identified that advanced age, female sex, nonfarmer occupation, higher income, higher education level, smoking, alcohol consumption, prior cardiovascular events, diabetes, and obesity were associated with higher risk of TC greater than or equal to 240 mg/dL, LDL-C greater than or equal to 160 mg/dL, or TG greater than or equal to 200 mg/dL. However, younger age, male sex, nonfarmer occupation, higher income, higher education level, smoking, no alcohol consumption, prior cardiovascular events, diabetes, and obesity were associated with higher risk of HDL-C less than 40 mg/dL (Table 2).

**Table 2.** Prevalence of Different Components of Dyslipidemia and Associated Characteristics

Characteristic	Overall dyslipidemia		TC ≥240 mg/dL		LDL-C ≥160 mg/dL		HDL-C <40 mg/dL		TG ≥200 mg/dL	
	Prevalence, % (95% CI)	OR (95% CI)^a^	Prevalence, % (95% CI)	OR (95% CI)^a^	Prevalence, % (95% CI)	OR (95% CI)^a^	Prevalence, % (95% CI)	OR (95% CI)^a^	Prevalence, % (95% CI)	OR (95% CI)^a^
Age, y										
35-44	29.95 (29.79-30.10)	1 [Reference]	3.10 (3.04-3.16)	1 [Reference]	1.75 (1.70-1.79)	1 [Reference]	18.64 (18.51-18.78)	1 [Reference]	14.71 (14.58-14.83)	1 [Reference]
45-54	32.69 (32.58-32.80)	1.17 (1.15-1.18)	6.17 (6.11-6.22)	2.14 (2.09-2.20)	3.40 (3.36-3.44)	2.11 (2.04-2.18)	16.36 (16.27-16.44)	0.89 (0.88-0.91)	17.45 (17.36-17.53)	1.21 (1.19-1.23)
55-64	34.10 (33.99-34.21)	1.21 (1.20-1.22)	8.80 (8.73-8.87)	3.23 (3.15-3.31)	5.01 (4.95-5.06)	3.20 (3.10-3.30)	14.90 (14.82-14.99)	0.76 (0.75-0.77)	17.88 (17.79-17.97)	1.21 (1.19-1.23)
65-75	31.86 (31.73-31.99)	1.07 (1.06-1.09)	8.51 (8.43-8.58)	3.24 (3.15-3.33)	4.73 (4.67-4.79)	3.13 (3.02-3.23)	14.12 (14.02-14.22)	0.67 (0.66-0.68)	15.85 (15.75-15.95)	1.05 (1.03-1.06)
Sex										
Male	36.67 (36.57-36.77)	1 [Reference]	4.06 (4.02-4.10)	1 [Reference]	2.62 (2.59-2.65)	1 [Reference]	24.35 (24.26-24.44)	1 [Reference]	16.60 (16.52-16.68)	1 [Reference]
Female	29.80 (29.72-29.88)	0.74 (0.73-0.74)	9.06 (9.01-9.11)	2.83 (2.78-2.88)	4.84 (4.81-4.88)	2.16 (2.12-2.21)	.01 (9.96-10.06)	0.30 (0.29-0.30)	16.97 (16.91-17.03)	1.16 (1.15-1.17)
Marital status										
Not married	32.27 (32.01-32.52)	1 [Reference]	9.50 (9.34-9.66)	1 [Reference]	4.98 (4.86-5.1)	1 [Reference]	12.32 (12.14-12.50)	1 [Reference]	17.67 (17.47-17.88)	1 [Reference]
Married	32.56 (32.50-32.62)	0.99 (0.98-1.01)	6.92 (6.88-6.95)	0.92 (0.90-0.94)	3.90 (3.87-3.92)	0.95 (0.93-0.98)	15.93 (15.88-15.98)	1.07 (1.05-1.09)	16.80 (16.74-16.85)	0.99 (0.97-1.00)
Annual household income, yuan^b^										
≤50 000	31.83 (31.76-31.90)	1 [Reference]	6.95 (6.92-6.99)	1 [Reference]	3.78 (3.75-3.81)	1 [Reference]	15.16 (15.11-15.22)	1 [Reference]	16.56 (16.50-16.62)	1 [Reference]
>50 000	35.79 (35.63-35.94)	1.07 (1.06-1.08)	7.59 (7.50-7.67)	1.02 (1.00-1.04)	4.64 (4.57-4.70)	1.06 (1.04-1.09)	18.07 (17.95-18.2)	1.08 (1.06-1.09)	18.53 (18.41-18.66)	1.07 (1.05-1.08)
Education level										
Lower than college	32.17 (32.11-32.24)	1 [Reference]	7.23 (7.19-7.26)	1 [Reference]	4.01 (3.98-4.04)	1 [Reference]	15.14 (15.09-15.19)	1 [Reference]	16.73 (16.68-16.78)	1 [Reference]
College or above	36.80 (36.57-37.03)	1.07 (1.06-1.09)	5.32 (5.21-5.42)	1.00 (0.98-1.03)	3.37 (3.28-3.45)	1.05 (1.02-1.08)	22.15 (21.96-22.35)	1.09 (1.07-1.11)	18.34 (18.16-18.52)	1.07 (1.05-1.09)
Occupation										
Not farmer	34.92 (34.83-35.00)	1 [Reference]	7.33 (7.28-7.37)	1 [Reference]	4.26 (4.22-4.29)	1 [Reference]	17.58 (17.51-17.65)	1 [Reference]	18.07 (18.00-18.13)	1 [Reference]
Farmer	29.88 (29.79-29.97)	0.87 (0.86-0.88)	6.76 (6.72-6.81)	0.93 (0.91-0.95)	3.62 (3.58-3.65)	0.89 (0.87-0.92)	13.68 (13.61-13.74)	0.84 (0.83-0.86)	15.43 (15.36-15.5)	0.88 (0.87-0.9)
Health insurance status										
Insured	32.54 (32.48-32.60)	1 [Reference]	7.08 (7.05-7.12)	1 [Reference]	3.96 (3.94-3.99)	1 [Reference]	15.69 (15.64-15.74)	1 [Reference]	16.86 (16.81-16.91)	1 [Reference]
Uninsured	34.78 (33.69-35.86)	1.02 (0.96-1.09)	7.35 (6.76-7.95)	0.94 (0.84-1.05)	3.85 (3.41-4.29)	0.93 (0.82-1.07)	16.42 (15.58-17.27)	1.05 (0.97-1.13)	18.59 (17.71-19.48)	1.00 (0.92-1.07)
CVD risk factor										
Current smoker	37.89 (37.74-38.03)	1.21 (1.2-1.23)	4.49 (4.42-4.55)	1.03 (1.00-1.05)	2.79 (2.74-2.84)	1.05 (1.02-1.08)	24.68 (24.55-24.81)	1.30 (1.29-1.32)	17.87 (17.76-17.99)	1.16 (1.15-1.18)
Current drinker	35.01 (34.88-35.14)	0.94 (0.93-0.95)	5.70 (5.64-5.76)	1.19 (1.17-1.21)	3.30 (3.25-3.35)	1.1 (1.08-1.13)	19.44 (19.33-19.55)	0.76 (0.75-0.77)	18.04 (17.94-18.15)	1.13 (1.12-1.15)
Diabetes	44.65 (44.39-44.91)	1.60 (1.58-1.62)	9.47 (9.32-9.62)	1.13 (1.11-1.15)	5.37 (5.25-5.48)	1.11 (1.08-1.14)	21.08 (20.87-21.29)	1.53 (1.51-1.56)	26.37 (26.14-26.6)	1.74 (1.71-1.76)
Obesity^c^	45.21 (45.05-45.38)	1.95 (1.94-1.97)	8.27 (8.18-8.37)	1.12 (1.10-1.14)	4.69 (4.62-4.76)	1.14 (1.12-1.16)	23.26 (23.12-23.40)	2.05 (2.03-2.07)	26.07 (25.93-26.22)	2.01 (1.99-2.03)
Prior CVD	38.94 (38.58-39.31)	1.15 (1.13-1.17)	7.71 (7.51-7.91)	1.00 (0.97-1.03)	4.52 (4.37-4.68)	1.08 (1.04-1.13)	19.78 (19.49-20.08)	1.24 (1.21-1.27)	20.90 (20.6-21.20)	1.08 (1.06-1.11)
Geographical region										
Western	32.58 (32.47-32.69)	1 [Reference]	5.24 (5.19-5.29)	1 [Reference]	2.94 (2.90-2.98)	1 [Reference]	18.92 (18.83-19.01)	1 [Reference]	15.52 (15.44-15.60)	1 [Reference]
Central	31.7 (31.58-31.81)	0.92 (0.90-0.95)	6.46 (6.40-6.53)	1.41 (1.34-1.48)	3.19 (3.14-3.23)	1.36 (1.28-1.43)	14.41 (14.32-14.50)	0.71 (0.68-0.74)	17.69 (17.59-17.78)	1.05 (1.02-1.08)
Eastern	33.16 (33.06-33.26)	0.98 (0.96-1.01)	9.19 (9.13-9.25)	1.90 (1.82-1.98)	5.47 (5.42-5.52)	2.09 (1.99-2.20)	13.84 (13.76-13.91)	0.77 (0.75-0.8)	17.36 (17.28-17.44)	0.95 (0.93-0.98)

Abbreviations: CVD, cardiovascular disease; HDL-C, high-density lipoprotein cholesterol; LDL-C, low-density lipoprotein cholesterol; OR, odds ratio; TC, total cholesterol; TG, triglycerides.

SI conversion factors: To convert HDL-C to millimoles per liter, multiply by 0.0259; LDL-C to millimoles per liter, multiply by 0.0259; TC to millimoles per liter, multiply by 0.0259; TG to millimoles per liter, multiply by 0.0113.

^a^ ORs were derived from multivariable regression models and adjusted for all sociodemographic and clinical characteristics included in Table 1.

^b^ The average conversion rate in 2019 was 6.91 yuan to $1.00 US.

^c^ Obesity is defined as a body mass index (weight in kilograms divided by height in meters squared) greater than or equal to 28.

### Treatment and Control of LDL-C Among Participants With Established and High Risk of ASCVD

**Figure 2.**
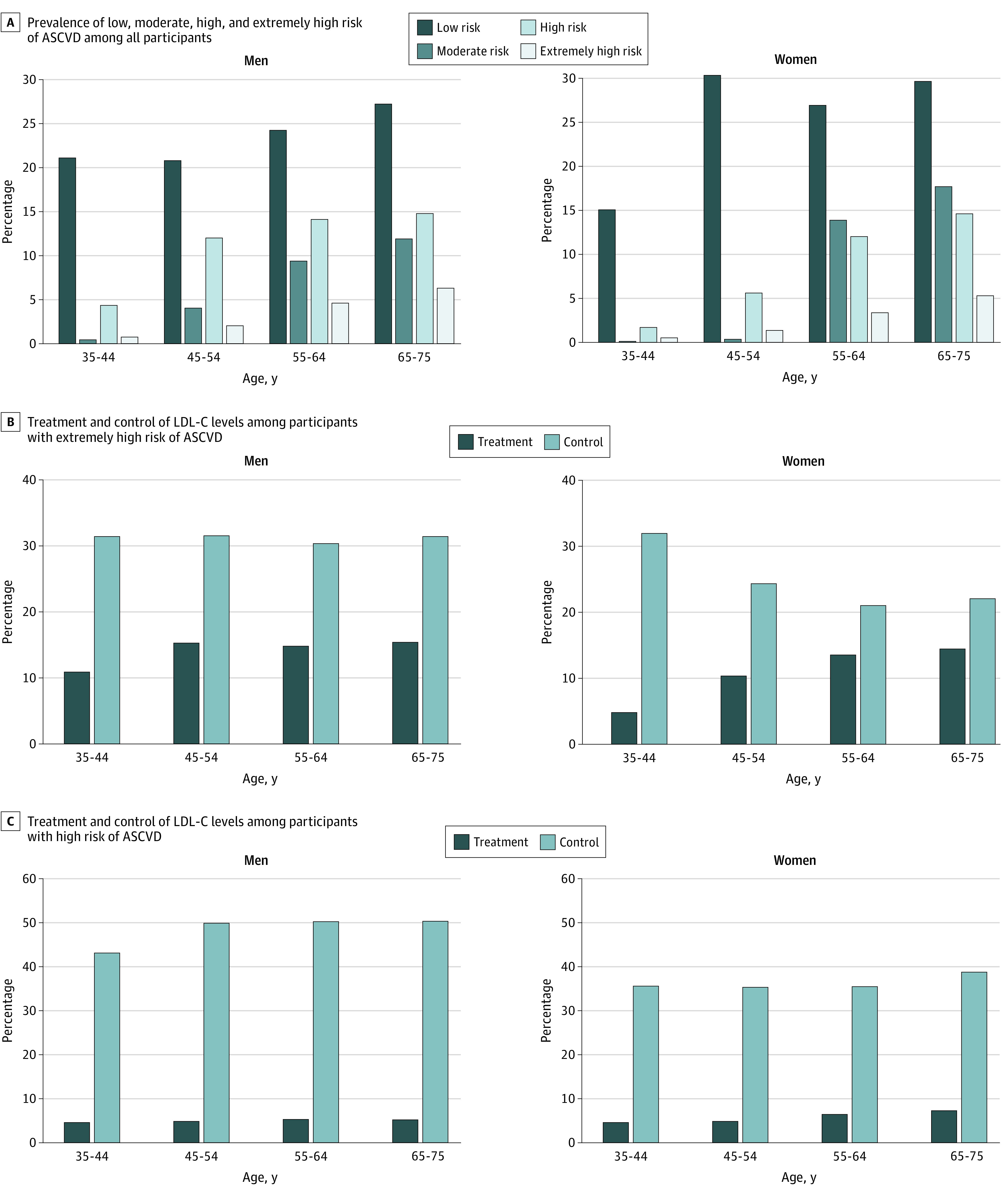
Prevalence, Treatment, and Low-Density Lipoprotein Cholesterol (LDL-C) Control of Participants With High or Extremely High Risk of Atherosclerotic Cardiovascular Disease (ASCVD) Panel A shows prevalence of low, medium, high and extremely high risk of ASCVD among all study participants. Panel B shows treatment and control of LDL-C among participants with extremely high risk of ASCVD. Panel C shows treatment and control of LDL-C among participants with high risk of ASCVD. Participants with extremely high risk of ASCVD were those with established ASCVD.

A total of 71 785 participants had established ASCVD and were recommended for lipid-lowering medications regardless of LDL-C levels, of whom 10 120 (14.1%) were treated and 61 665 (85.9%) were untreated with any lipid-lowering medications. The control rate of LDL-C (≤70 mg/dL) among adults with established ASCVD was 26.6% (19 087 participants), with the control rate being 44.8% (4535 participants) among those who were treated and 23.6% (14 552 participants) among those who were untreated (Figure 2).

A total of 236 579 participants had high risk of ASCVD, of whom 101 474 (42.9%) achieved LDL-C control targets (≤100 mg/dL). Among 135 105 participants who had high risk of ASCVD and LDL-C greater than 100 mg/dL, 6044 (4.5%) were treated with lipid-lowering medications (Figure 2). Among 769 722 participants with low or moderate risk of ASCVD, the overall treatment and control rates of LDL-C were 2.2% (17 087 participants) and 55.7% (428 588 participants), respectively.

In multivariable regression analysis, we identified that advanced age (age 65-75 years, odds ratio [OR], 0.63; 95% CI, 0.56-0.70), female sex (OR, 0.56; 95% CI, 0.53-0.58), lower income (reference category), smoking (OR, 0.89; 95% CI, 0.85-0.94), alcohol consumption (OR, 0.87; 95% CI, 0.83-0.92), and no diabetes (reference category) were associated with lower control of LDL-C among participants with established ASCVD. Younger age (reference category) and female sex (OR, 0.58; 95% CI, 0.56-0.59) were associated with lower control of LDL-C among participants with high risk of ASCVD (Table 3).

**Table 3.** Treatment Rates and Control Rate of LDL-C and Associated Characteristics by ASCVD Risk Groups

Characteristic	Treatment of LDL-C				Control of LDL-C			
	Participants with high risk of ASCVD		Participants with established ASCVD		Participants with high risk of ASCVD		Participants with established ASCVD	
	Prevalence, % (95% CI)	OR (95% CI)^a^	Prevalence, % (95% CI)	OR (95% CI)^a^	Prevalence, % (95% CI)	OR (95% CI)^a^	Prevalence, % (95% CI)	OR (95% CI)^a^
Age, y								
35-44	4.51 (4.09-4.94)	1 [Reference]	7.88 (6.73-9.03)	1 [Reference]	40.24 (39.23-41.25)	1 [Reference]	31.76 (29.76-33.75)	1 [Reference]
45-54	4.72 (4.54-4.89)	1.10 (0.99-1.24)	12.79 (12.17-13.4)	1.47 (1.24-1.75)	43.55 (43.14-43.96)	1.15 (1.09-1.22)	27.75 (26.93-28.57)	0.74 (0.66-0.83)
55-64	5.80 (5.65-5.95)	1.34 (1.20-1.50)	14.13 (13.71-14.54)	1.47 (1.25-1.74)	41.84 (41.52-42.17)	1.11 (1.06-1.17)	25.4 (24.88-25.92)	0.62 (0.56-0.70)
65-75	6.27 (6.09-6.44)	1.49 (1.33-1.66)	14.73 (14.32-15.14)	1.46 (1.23-1.72)	43.88 (43.52-44.24)	1.19 (1.13-1.26)	26.52 (26.01-27.04)	0.63 (0.56-0.70)
Sex								
Male	5.02 (4.89-5.15)	1 [Reference]	14.96 (14.58-15.35)	1 [Reference]	49.81 (49.51-50.11)	1 [Reference]	30.99 (30.5-31.49)	1 [Reference]
Female	6.19 (6.05-6.33)	1.30 (1.25-1.35)	13.04 (12.69-13.39)	0.86 (0.82-0.91)	36.47 (36.19-36.74)	0.58 (0.56-0.59)	22.22 (21.79-22.65)	0.56 (0.53-0.58)
Marital status								
Not married	5.89 (5.54-6.25)	1 [Reference]	13.35 (12.49-14.20)	1 [Reference]	41.95 (41.21-42.7)	1 [Reference]	24.29 (23.21-25.37)	1 [Reference]
Married	5.63 (5.53-5.73)	1 (0.93-1.07)	14.05 (13.78-14.33)	1.08 (0.99-1.17)	42.95 (42.74-43.16)	1 (0.96-1.03)	26.69 (26.34-27.04)	1.02 (0.95-1.09)
Annual household income, yuan^b^								
≤50 000	5.02 (4.91-5.13)	1 [Reference]	12.81 (12.53-13.09)	1 [Reference]	43.05 (42.81-43.29)	1 [Reference]	26.41 (26.04-26.78)	1 [Reference]
>50 000	7.91 (7.65-8.16)	1.35 (1.28-1.41)	21.61 (20.79-22.44)	1.25 (1.17-1.34)	42.94 (42.47-43.41)	1.01 (0.98-1.04)	27.4 (26.51-28.3)	1.09 (1.03-1.17)
Education level								
Lower than college	5.47 (5.37-5.57)	1 [Reference]	13.63 (13.36-13.89)	1 [Reference]	42.71 (42.49-42.92)	1 [Reference]	26.37 (26.03-26.72)	1 [Reference]
College or above	7.96 (7.53-8.39)	1.36 (1.27-1.46)	19.39 (18.22-20.55)	1.29 (1.18-1.42)	45.34 (44.55-46.13)	0.96 (0.92-1.00)	27.78 (26.46-29.1)	1.04 (0.95-1.13)
Occupation								
Not farmer	6.65 (6.52-6.78)	1 [Reference]	16.71 (16.33-17.08)	1 [Reference]	42.92 (42.65-43.19)	1 [Reference]	25.27 (24.84-25.71)	1 [Reference]
Farmer	4.21 (4.09-4.34)	0.68 (0.65-0.71)	10.59 (10.25-10.93)	0.72 (0.67-0.76)	42.74 (42.43-43.06)	0.98 (0.95-1.01)	27.88 (27.38-28.38)	1.00 (0.95-1.06)
Health insurance status								
Insured	5.63 (5.54-5.73)	1 [Reference]	13.98 (13.72-14.24)	1 [Reference]	42.86 (42.65-43.06)	1 [Reference]	26.44 (26.11-26.77)	1 [Reference]
Uninsured	4.19 (2.64-5.74)	0.76 (0.51-1.14)	8.23 (3.94-12.51)	0.68 (0.37-1.25)	37.73 (33.99-41.48)	0.91 (0.75-1.10)	22.78 (16.24-29.33)	1.09 (0.69-1.71)
Cardiovascular disease risk factor								
Current smoker	5.02 (4.86-5.18)	NA	13.22 (12.68-13.77)	0.87 (0.82-0.93)	49.21 (48.84-49.57)	NA	28.87 (28.14-29.6)	0.89 (0.85-0.94)
Current drinker	5.66 (5.48-5.84)	NA	14.70 (14.15-15.26)	0.95 (0.89-1.01)	47.17 (46.79-47.55)	NA	27.28 (26.58-27.98)	0.87 (0.83-0.92)
Diabetes	7.19 (7.05-7.33)	NA	22.36 (21.58-23.13)	1.68 (1.59-1.78)	52.9 (52.62-53.17)	NA	29.22 (28.37-30.07)	1.22 (1.15-1.28)
Obesity	6.94 (6.73-7.15)	NA	16.66 (16.06-17.26)	1.23 (1.17-1.30)	44.60 (44.19-45)	NA	25.99 (25.28-26.7)	0.99 (0.95-1.04)
Geographical region								
Western	4.23 (4.07-4.38)	1 [Reference]	12.10 (11.65-12.55)	1 [Reference]	48.05 (47.66-48.44)	1 [Reference]	28.74 (28.12-29.37)	1 [Reference]
Eastern	5.23 (5.05-5.40)	1.35 (1.28-1.43)	12.48 (12.08-12.87)	1.24 (1.12-1.37)	45.24 (44.84-45.63)	0.77 (0.74-0.81)	26.60 (26.07-27.13)	0.87 (0.81-0.93)
Central	6.73 (6.58-6.88)	1.54 (1.47-1.62)	17.51 (17.00-18.01)	1.81 (1.65-1.99)	38.24 (37.94-38.54)	0.58 (0.55-0.61)	24.13 (23.57-24.7)	0.76 (0.71-0.82)

Abbreviations: ASCVD, atherosclerotic cardiovascular disease; LDL-C, Low-density lipoprotein cholesterol; NA, not applicable; OR, odds ratio.

^a^ ORs were derived from multivariable regression models and adjusted for all sociodemographic and clinical characteristics included in Table 1. We did not include smoking, alcohol use, obesity, and diabetes for model among participants with high risk of ASCVD because these variables were used to calculate risk of ASCVD.

^b^ The average conversion rate in 2019 was 6.91 yuan to $1.00 US.

### Availability of Lipid-Lowering Medications in Primary Care Institutions

Of the 3529 primary care institutions surveyed, 3041 with completed medication availability data were included in the final analysis. These institutions included 145 community health centers and 384 community health stations from the urban areas, 243 township health centers and 2269 village clinics from the rural areas (eTable 1 in Supplement 1).

Of 3041 primary care institutions included in the analysis, 1512 (49.7%) stocked statins, 584 (19.2%) stocked nonstatins, and 467 (15.4%) stocked lipid-lowering TCM. Xuezhikang, the only TCM that had evidence of secondary prevention for CVD, was stocked in 311 primary care institutions (10.2%). Among the 4 types of institutions, community health centers had the highest statin availability (114 centers [78.6%]), whereas village clinics had the lowest statin availability (991 clinics [43.7%]) (eFigure 3 in Supplement 1). Simvastatin was the most commonly stocked statin (1422 clinics [46.8%]), followed by atorvastatin (808 clinics [26.6%]), and rosuvastatin (559 clinics [18.4%]) (eTable 2 in Supplement 1).

## Discussion

This large, national cross-sectional study found that dyslipidemia is highly prevalent in China but commonly undertreated and uncontrolled. Even among people with established ASCVD and high risk of ASCVD, only 26.6% and 42.9%, respectively, achieved LDL-C control targets. Moreover, statins, the evidence-based lipid-lowering medications recommended by the guideline, are not available in almost one-half of the primary care institutions, with the lowest availability in rural village clinics.

Our study extends the literature in several important ways. First, our study is one of the largest and most recent studies to show the nationwide characteristics of the dyslipidemia epidemic in China. Although the prevalence of dyslipidemia was consistent with previous studies and meta-analyses,^4,5,13,14,15^ the large size of our study allowed us to draw robust conclusions across a wide variety of subgroups. Our results reveal that dyslipidemia has become a major factor associated with the risk of CVD in China overall and across diverse population subgroups, suggesting that a national approach is warranted to mitigate dyslipidemia and the resulting burden of CVD. In addition, low HDL-C and high TG levels have become the dominant components of dyslipidemia, which are different than the high TC and LDL-C levels found in the US and European countries.^16,17,18^ This finding calls for attention to HDL-C and TG management in addition to LDL-C control emphasized in most of the current guidelines. Icosapent ethyl, a new TG-lowering drug, has been shown to have a substantial benefit with respect to major adverse cardiovascular events in the recent REDUCE-IT trial^19,20^ and was granted priority review by the US Food and Drug Administration.^21^ China may consider adopting new treatments to control TG as they become available. Moreover, lifestyle modifications endorsed by guidelines to improve HDL-C and TG may be particularly relevant to the Chinese population and should be promoted.^10^

Second, we showed that despite the low prevalence of increased LDL-C, the absolute number of people with elevated LDL-C is still large, yet its treatment and control are far from optimal. In particular, for patients with established ASCVD, statins were recommended as first-line medications for risk reduction irrespective of LDL-C levels according to the secondary prevention guidelines.^22,23^ However, only 14.1% of patients with established ASCVD in our study were treated with lipid-lowering medications and the use of statins was even lower. We further identified that younger age and female sex were associated with lower control of LDL-C among people with high risk of ASCVD, indicating that preventive interventions to control LDL-C should be targeted to these subgroups for optimal outcomes.

Third, to our knowledge, this study is the first national study to assess the availability of lipid-lowering medications in primary care institutions from all 31 provinces in China. Previous studies were limited to specific regions, populations, or data sources.^13,24,25,26,27,28,29^ We found that evidence-based medications, such as statins, were stocked in only one-half of the primary care institutions. One of the commonly stocked TCMs, xuezhikang, was endorsed by the Chinese guideline^10^ because studies showed it had a lipid-lowering effect similar to that of statins.^30,31,32^ However, the safety and efficacy of other TCMs stocked in primary care institutions were unclear and need additional evaluation.

Our study found that the associations between different forms of dyslipidemia and some risk factors, including age, sex, and alcohol consumption, may be different. The differential association may be associated with different sex hormones in men and women. It has been shown that menopause leads to changes in lipid profile through reducing HDL-C and increasing TC, TG, and LDL-C.^33^ Our study also supports a previous study^34^ reporting that lower alcohol consumption was associated with higher HDL-C levels. Studies^35^ have shown that alcohol reduces the activity of cholesterol ester transformation from HDL to atheromatic molecules, subsequently increased the circulating levels of HDL-C.

Our study has important policy implications. Despite historically low lipid levels in the Chinese population, marked changes in diet and physical activity, especially increased dietary energy intake and sedentary behaviors, has increased the rate of dyslipidemia, a factor substantially associated with the risk of CVD.^36,37,38^ If China seeks to mitigate increasing prevalence of CVD, reducing dyslipidemia is a potential good target. An important step is to increase the capacity of primary care institutions to screen, diagnose, and treat dyslipidemia in community residents. The Chinese health reform in 2009 emphasized the role of primary health care as the gatekeeper to health care system.^39^ However, routine screening programs for blood lipid levels are still not provided in primary care institutions, limiting the detection and treatment of dyslipidemia. In addition, it is important to ensure that effective lipid-lowering medications are available in primary care institutions where basic medical services are provided. The marked deficiencies in statin availability at primary care institutions are not consistent with the health needs of the people and have implications for patients’ health. The 2018 National Essential Medicines List^40^ has included more evidence-based lipid-lowering medications and could facilitate the provision of these medications; however, this program is in the early stages of implementation. Finally, effective community-based prevention strategies that promote lifestyle modification (eg, regular physical activity and dietary improvement) are needed to control dyslipidemia, particularly HDL-C and TG levels, in the Chinese population.

### Limitations

Our study had some limitations. First, our study used a purposeful, rather than random, sampling strategy. However, participants from all ethnic groups across all provinces in China were included, and the ratios of participants in rural to urban areas in each province were comparable to national census data. Compared with the age and sex distribution of national census data, this study has more women and more adults in the older age group, which may result in overestimating the prevalence of dyslipidemia. However, we calculated the age- and sex-standardized estimates for dyslipidemia prevalence according to the 2010 Chinese Census data, and the estimates closely resemble our main results. In addition, use of lipid-lowering medications was self-reported, which could be subject to recall bias. We asked a subset of participants to bring in the bottle of mediations for validation purpose and found that self-reported data may tend to underestimate the overall treatment rate, because some participants who received treatment could not recall the name of lipid-lowering medications. Nevertheless, ongoing rates of dyslipidemia in those taking lipid-lowering medications reflect that it is likely few were taking effective medications. Furthermore, we defined diabetes according to physician diagnosis only, which tends to underestimate the overall prevalence of diabetes given the large proportion of people with undiagnosed diabetes. Because people with diabetes are considered at high risk of ASCVD and require lipid-lowering therapy for risk reduction, our analysis tends to underestimate the proportion of participants with high risk of ASCVD.

## Conclusions

In conclusion, the findings of this study suggest that dyslipidemia has become a major public health problem in China and is often inadequately treated and uncontrolled. Statins were available in less than one-half of the primary care institutions, with the lowest availability in rural village clinics. A focus on lipid management, in addition to other emerging risk factors, including hypertension, smoking, and air quality, represents immense opportunities for China to stem an increasing population threat of cardiovascular diseases.

## Notes

Supplement 1. **eAppendix 1.** Detailed Information About Sampling Selection in China-PEACE Million Persons Project **eAppendix 2.** List of Lipid-Lowering Medications **eAppendix 3.** Classification of Low, Medium, High, and Extremely High Risk of ASCVD According to Chinese Guideline **eTable 1.** Characteristics of Primary Care Institutions Included in the Analysis **eTable 2.** Individual Drugs Available in the Primary Care Institutions **eFigure 1.** Flowchart of Study Participant Selection in China-PEACE Million Persons Project **eFigure 2.** Distribution of Lipid Profile Among Participants in China-PEACE Million Persons Project **eFigure 3.** Availability of Lipid-Lowering Medications (Statin, Non-Statin, Traditional Chinese Medicine) Among 3,041 Primary Care Institutions, by Type of Site and Economic Region

Supplement 2. **China Patient-Centered Evaluative Assessment of Cardiac Events **M**illion Persons Project (China-PEACE MPP) Collaborative Group Members**
