# Supplementary material for: Prevalence of Dyslipidemia and Availability of Lipid-Lowering Medications Among Primary Health Care Settings in China
Source: JAMA Netw Open. 2021 Sep 29;4(9):e2127573. doi: 10.1001/jamanetworkopen.2021.27573 (PMC8482054; doi:10.1001/jamanetworkopen.2021.27573)
Supplement: Supplement 1. — eAppendix 1. Detailed Information About Sampling Selection in China-PEACE Million Persons Project eAppendix 2. List of Lipid-Lowering Medications eAppendix 3. Classification of Low, Medium, High, and Extremely High Risk of ASCVD According to Chinese Guideline eTable 1. Characteristics of Primary Care Institutions Included in the Analysis eTable 2. Individual Drugs Available in the Primary Care Institutions eFigure 1. Flowchart of Study Participant Selection in China-PEACE Million Persons Project eFigure 2. Distribution of Lipid Profile Among Participants in China-PEACE Million Persons Project eFigure 3. Availability of Lipid-Lowering Medications (Statin, Non-Statin, Traditional Chinese Medicine) Among 3,041 Primary Care Institutions, by Type of Site and Economic Region [file jamanetwopen-e2127573-s001.pdf]

Supplemental Online Content

Lu Y, Zhang H, Lu J, et al; for the China Patient-Centered Evaluative Assessment of Cardiac Events Million Persons Project Collaborative Group. Prevalence of dyslipidemia and availability of lipid-lowering medications among primary health care settings in China. *JAMA Netw Open*. 2021;4(9):e2127573.
doi:10.1001/jamanetworkopen.2021.27573

eAppendix 1. Detailed Information About Sampling Selection in China-PEACE Million Persons Project
eAppendix 2. List of Lipid-Lowering Medications
eAppendix 3. Classification of Low, Medium, High, and Extremely High Risk of ASCVD According to Chinese Guideline
eTable 1. Characteristics of Primary Care Institutions Included in the Analysis
eTable 2. Individual Drugs Available in the Primary Care Institutions
eFigure 1. Flowchart of Study Participant Selection in China-PEACE Million Persons Project
eFigure 2. Distribution of Lipid Profile Among Participants in China-PEACE Million Persons Project
eFigure 3. Availability of Lipid-Lowering Medications (Statin, Non-Statin, Traditional Chinese Medicine) Among 3,041 Primary Care Institutions, by Type of Site and Economic Region

This supplemental material has been provided by the authors to give readers additional information about their work.

© 2021 Lu Y et al. *JAMA Network Open.*

eAppendix 1. Detailed Information About Sampling Selection in China-PEACE Million Persons Project

From September 2014 to November 2018, we selected 189 sites (114 rural counties, 75 urban districts) from all 31 provinces based on their geographic locations within each province, number of residents live in rural or urban area, minority ethnicity distribution, quality of disease and death registries, and local capacity to support the project (see **Figure** below). Specifically, staff in provincial coordinating office collected basic information (geographic information, economic development, population size, and minority ethnicity distribution) about the selected sites in their provinces; reported this information to the national coordinating office; discussed with staff in the national coordinating office to determine the study sites. In each site, about 5 towns or sub-districts were chosen according to their size, population stability (e.g., no sudden significant change in the number of residents), local workers' commitment, and the ability to perform the screening. Initial screening stations were set up in each town or subdistrict health center.

Potentially eligible participants were identified in each town or sub-district through official residential records and then invited by local community workers via telephone or extensive publicity campaigns on television and in newspapers. All participants were required to bring their identity cards to the screening center to verify that they met both inclusion criteria: 1) aged 35 to 75 years; 2) registered in the selected site's Hukou (a record officially identifying a person as a resident of an area), and residents lived in the selected regions at least 6 months during the last 12 months were enrolled during 2014–2018. After their residency was verified, participants who had signed the informed consent agreement were then enrolled in the project.

Figure. Study sites in China-PEACE Million Persons Project

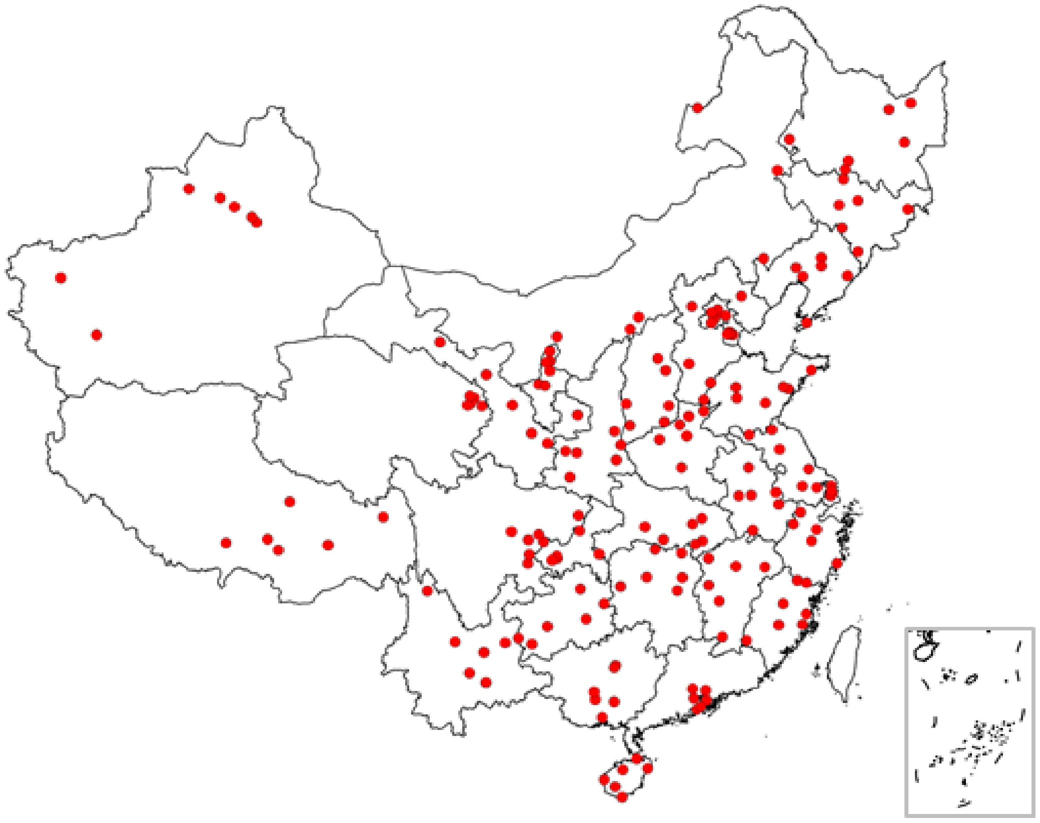

© 2021 Lu Y et al. *JAMA Network Open.*

eAppendix 2. List of Lipid-Lowering Medications

Classes	Name
**Statins**	Simvastatin
	Atorvastatin
	Rosuvastatin
	Lovastatin
	Fluvastatin
	Pravastatin
	Pitavastatin
**Non-statins**	Clofibric Acid
	Etofylline Clofibrate
	Calcium Clofibrate
	Magnesium Clofibrate
	Lifibrate
	Simfibrate
	Gemfibrozil
	Aluminum Clofibrate
	Ciprofibrate
	Fenofibrate
	Dextran Sulfate
	Clofibrate
	Bezafibrate
	Psyllium Hydrophilic Mucilloid
	Colestipol
	Coleselan hydrochloride
	Colestipol Hydrochloride
	Cholestyramine
	Hyodeoxycholic Acid
	Compound Trivitamin and Linolic Acid Soft
	Compound Calcium Linoleic Acid
	ω-3 Fatty Acids
	Compound Ethyl Linoleate Soft
	Ethyl Polyenoate
	Bombyx Mori Oil Ethyl Linolenate
	Ethyl Linoleate
	Garlic Oil
	Disodium Glycyrrhetate
	Cinametic Acid
	Lomitapide
	Febuprol
	Chondroitin Sulfate
	Mucopolysaccharide
	Diisopropylamine Ascorbate
	Sitosterol
	Pyricarbate
	Probucol
	Lomitapide mesylate
	Mibomesen Sodium
	Alginidatum Natricum
	Alginic Sodium Diester and Sodium Chloride
	Compound Calcium Linoleic Acid

	furazabol	
	Chondroitin Sulfate A Sodium	
	Mipomersen	
	Nafenopin	
	Myricin	
	Chondroitin	
	Mannose Ester	
	Eicosapentaenoic Acid	
	Linoleic Acid	
	Policosanol	
	Qsalmide	
	Polysaccharide Esters	
	ω-3-Acid Ethyl Ester	
	Ezetimibe	
	Carnitine	
	Allitride	
	Elastase	
	Dextran Sulfate	
	Chondroitin Sulfate A	
	Dihydroxydibutylether	
	Acidum Linolcicum Vitamium E /Two Vitamium Rutinum Tablets	
	Melinamide	
	Chondroitin Sulfate A	
	Fenofibric Acid	
	Pantethine	
	Dioscin	
	Chondroitin Sulfate Sodium	
	Vitmain E and Three Oils Soft	
	Metreleptin	
	Inositol	
	Niacin	
	Mannitole Nicotinate	
	Nicotinic Acid	
	Acipimox	
	Inositoli Nicotinas	
**Lipid-lowering traditional Chinese medicines**	**Trade name in Chinese**	**Trade name in English**
	藏降脂胶囊	Tibetan lipid-lowering capsules
	丹田降脂丸	Dantian lipid-lowering pills
	丹香清脂颗粒	Danxiangqingzhi lipid granule
	调脂胶囊	TiaoZhi capsules
	调脂片	Tiaozhi tablets
	调脂清肝胶囊	Tiaozhi qinggan capsules
	复方花粉调脂胶囊	Compound pollen tiaozhi capsules
	复方降脂胶囊	Compound tiaozhi capsules
	复方降脂片	Compound tiaozhi tablets
	葛山降脂颗粒	Geshan jiangzhi granules
	荷叶调脂茶	Lotus leaf lipid-lowering tea

© 2021 Lu Y et al. *JAMA Network Open.*

	健脾降脂颗粒	Spleen-fortifying lipid-lowering granules
	姜黄清脂胶囊	Turmeric qingzhi capsules
	姜黄清脂片	Turmeric qingzhi tablets
	姜黄清脂丸	Turmeric qingzhi pills
	降糖消脂胶囊	Glucose-lowering xiaozhi capsules
	降脂茶	Lipid-lowering tea
	降脂化浊胶囊	Lipid-lowering and turbidity-resolving capsules
	降脂减肥胶囊	Lipid-reducing slimming capsules
	降脂减肥片	Lipid-reducing slimming tablets
	降脂颗粒	Lipid-lowering granules
	降脂灵分散片	Lipid-lowering dispersible tablets
	降脂灵胶囊	Jiangzhiling capsules
	降脂灵颗粒	Jiangzhiling granules
	降脂灵片	Jiangzhiling tablets
	降脂宁胶囊	Jiangzhining capsules
	降脂宁颗粒	Jiangzhining granules
	降脂宁颗粒(无糖型)	Jiangzhining granules (sugar-free type)
	降脂宁片	Jiangzhining tablets
	降脂排毒胶囊	Lipid-lowering and detoxification capsules
	降脂平口服液	Jiangzhiping oral solution
	降脂软胶囊	Lipid-lowering soft capsules
	降脂通便胶囊	Lipid-lowering and tong-bian capsules
	降脂通络软胶囊	Lipid-lowering and tongluo soft capsules
	降脂通脉胶囊	Jiangzhi tongmai capsules
	降脂消食灵	Lipid-lowering and xiaoshiling
	降脂益肝胶囊	Jiangzhi yigan capsules
	解毒降脂胶囊	Detoxication and lipid-lowering capsules
	解毒降脂片	Detoxication and lipid-lowering tablets
	决明降脂胶囊	Cascade lipid-lowering capsules
	决明降脂片	Cascade lipid-lowering tablets
	决明平脂胶囊	Cascade pingzhi capsules
	蓝芪降脂合剂	Lanqi lipid-lowering mixture
	沥水调脂胶囊	Lishui tiaozhi capsules
	绿荷清脂茶	Green lotus lipid-lowering tea
	明藿降脂颗粒	Minghuo lipid-lowering granules

	排毒降脂胶囊	Detoxification and lipid-lowering capsules
	排毒清脂胶囊	Detoxification and qingzhi capsules
	排毒清脂颗粒	Detoxification and qingzhi granules
	排毒清脂片	Detoxification and qingzhi tablets
	排毒清脂软胶囊	Detoxification and qingzhi soft capsules
	清脂胶囊	Qingzhi capsules
	三参降脂液	Sanshen jiangzhi liquid solution
	桑葛降脂丸	Sangge jiangzhi pills
	沙棘脂康合剂	Seabuckthorn mixture
	山庄降脂颗粒	Shanzhuang jiangzhi granules
	山庄降脂片	Shanzhuang jiangzhi tablets
	山楂精降脂滴丸	Hawthorn refined lipid-lowering pills
	山楂精降脂分散片	Hawthorn refined lipid-lowering dispersible tablets
	山楂精降脂片	Hawthorn refined lipid-lowering tablets
	山楂精降脂软胶囊	Hawthorn refined lipid-lowering soft capsules
	舒心降脂片	Shuxin lipid-lowering tablets
	双勒调脂胶囊	Shuangle tiaozhi capsules
	泰脂安胶囊	Taizhi'an capsules
	糖脂宁胶囊	Tangzhining capsules
	糖脂消口服液	Tangzhixiao oral solution
	通脉降脂胶囊	Tongmai lipid-lowering capsules
	通脉降脂咀嚼片	Tongmai lipid-lowering chewable tablets
	通脉降脂颗粒	Tongmai lipid-lowering granules
	通脉降脂片	Tongmai lipid-lowering tablets
	通脉降脂丸	Tongmai lipid-lowering pills
	维脂康胶囊	Weizhikang capsules
	乌丹降脂颗粒	Wudan lipid-lowering granules
	乌杞调脂口服液	Wuyi tiaozhi oral solution
	五味降脂胶囊	Wuwei lipid-lowering capsules
	五子降脂胶囊	Wuzi jiangzhi capsules
	消瘀降脂胶囊	Xiaoyu lipid-lowering capsules
	血脂胶囊	Xuezhi capsules
	血脂康胶囊	Xuezhikang capsules
	血脂康片	Xuezhikang tablets
	血脂灵胶囊	Xuezhiling capsules

© 2021 Lu Y et al. *JAMA Network Open.*

	血脂灵颗粒	Xuezhiling granules
	血脂灵片	Xuezhiling tablets
	血脂宁丸	Xuezhining pills
	血脂平胶囊	Xuezhiping capsules
	血脂平软胶囊	Xuezhiping soft capsules
	益脂平胶囊	Yizhiping capsules
	泽术降脂胶囊	Zeshu lipid-lowering capsules
	真菌降脂素胶囊	Fungal lipid-lowering capsules
	正心降脂片	Zhengxin lipid-lowering tablets
	脂必泰胶囊	Zhibitai capsules
	脂必妥胶囊	Zhibituo capsules
	脂必妥咀嚼片	Zhibituo chewable tablets
	脂必妥片	Zhibituo tablets
	脂衡颗粒	Zhiheng granules
	脂降宁片	Zhijiangning tablets
	脂康颗粒	Zhikang granules
	脂可清胶囊	Zhikeqing capsules
	脂脉康胶囊	Zhimaikang capsules
	脂清胶囊	Zhiqing capsules
	脂清片	Zhiqing tablets
	脂欣康颗粒	Zhixinkang granules
	紫苏降脂软胶囊	Perilla lipid-lowering soft capsules
	葶苈降血脂胶囊	Tingli blood lipid-lowering capsules
	葶苈降血脂颗粒	Tingli blood lipid-lowering granules
	葶苈降血脂片	Tingli blood lipid-lowering tablets
	楂明调脂颗粒	Zhaming tiaozhi granules

© 2021 Lu Y et al. *JAMA Network Open.*

eAppendix 3. Classification of Low, Medium, High, and Extremely High Risk of ASCVD According to Chinese Guideline

Those who meet any of the following conditions can be directly classified as high-risk or extremely high-risk groups

Extremely high risk: patients with established ASCVD

High risk: (1) LDL-C ≥4.9 mmol/L(189 mg/dl) or TC ≥7.2 mmol/L (278 mg/dl)

(2) patients with diabetes [LDL-C 1.8-4.9 mmol/L (70-189mg/dl) (or TC 3.1-7.2 mmol/L(120-189mg/dl)) and age ≥ 40 years]

Condition not met, evaluate the 10-year risk of ASCVD

Number of risk factor*		Classification of serum cholesterol levels, mmol/L(mg/dl)		
		3.1 (120)≤ TC < 4.1(159) Or 1.8(70) ≤ LDL-C < 2.6(100)	4.1(159) ≤ TC < 5.2(200) Or 2.6(100) ≤ LDL-C < 3.4(130)	5.2 (200)≤ TC < 7.2(278) Or 3.4(130) ≤ LDL-C < 4.9(189)
Without hypertension	0-1	Low risk (<5%)	Low risk (<5%)	Low risk (<5%)
	2	Low risk (<5%)	Low risk (<5%)	Medium risk (5%-9%)
	3	Low risk (<5%)	Medium risk (5%-9%)	Medium risk (5%-9%)
With hypertension	0	Low risk (<5%)	Low risk (<5%)	Low risk (<5%)
	1	Low risk (<5%)	Medium risk (5%-9%)	Medium risk (5%-9%)
	2	Medium risk (5%-9%)	High risk (≥10%)	High risk (≥10%)
	3	High risk (≥10%)	High risk (≥10%)	High risk (≥10%)

For people with medium 10-year risk of ASCVD and age <55, evaluate the following risk factors

Those who meet any two and more of the following conditions can be directly classified as high-risk group

High risk: (1) Systolic blood pressure ≥ 160 mmHg or diastolic blood pressure ≥ 100 mmHg

(2) Non-HDL-C ≥ 5.2 mmol/L (200 mg/dl)

(3) HDL-C < 1.0 mmol/L (40 mg/dl)

(4) BMI ≥ 28 kg/m^2

(5) Current smoking

Adapted from the 2016 Chinese Adult Dyslipidemia Prevention Guideline.[9]

*Risk factors include current smoking, low HDL-C and men with age ≥ 45 or women with age ≥ 55; patients with chronic kidney disease

ASCVD = atherosclerotic cardiovascular disease, TC= total cholesterol, LDL-C = low-density lipoprotein cholesterol, HDL-C = how-density lipoprotein cholesterol, BMI= body mass index.

© 2021 Lu Y et al. *JAMA Network Open.*

eTable 1. Characteristics of Primary Care Institutions Included in the Analysis

	Rural		Urban		Total
	Township health center	Village clinic	Community health center	Community health station	
**Primary health care site**					
N	243	2269	145	384	3041
Region (%)					
Eastern	32.1	30.2	49.7	58.1	34.8
Central	32.1	39.1	20.0	16.4	34.8
Western	35.8	30.7	30.3	25.5	30.5

© 2021 Lu Y et al. *JAMA Network Open.*

eTable 2. Individual Drugs Available in the Primary Care Institutions

Drug name	Availability percentage (%)
**Statin**	49.7
Simvastatin	46.8
Atorvastatin	26.6
Rosuvastatin	18.4
Lovastatin	4.4
Fluvastatin	1.1
Pravastatin	1.0
Pitavastatin	0.8
**Traditional Chinese medicine**	15.4
Xuezhikang capsules or tablets	10.2
Other TCM	6.2
**Non-statin**	19.2

© 2021 Lu Y et al. *JAMA Network Open.*

eFigure 1. Flowchart of Study Participant Selection in China-PEACE Million Persons Project

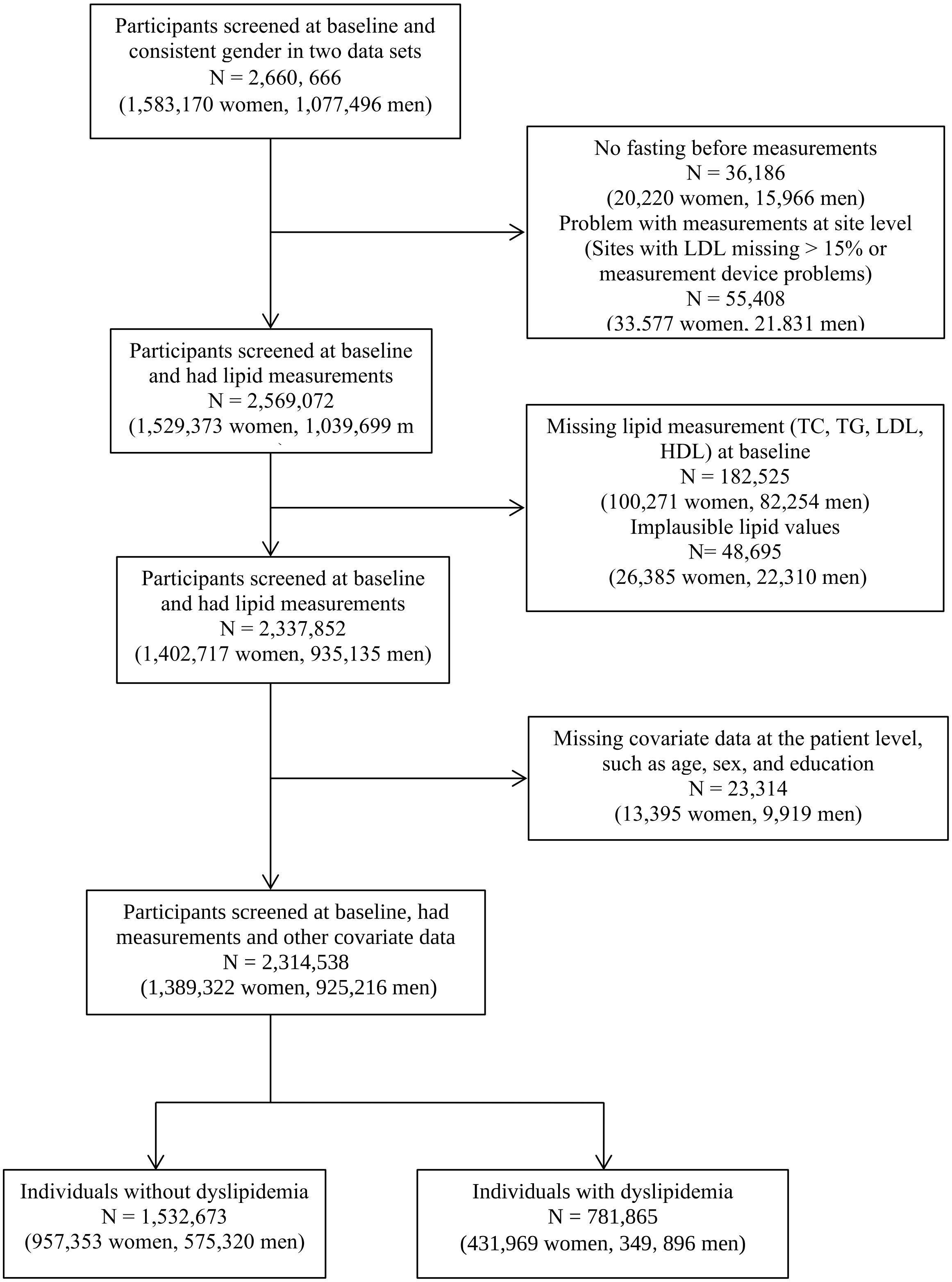

© 2021 Lu Y et al. *JAMA Network Open.*

eFigure 2. Distribution of Lipid Profile Among Participants in China-PEACE Million Persons Project

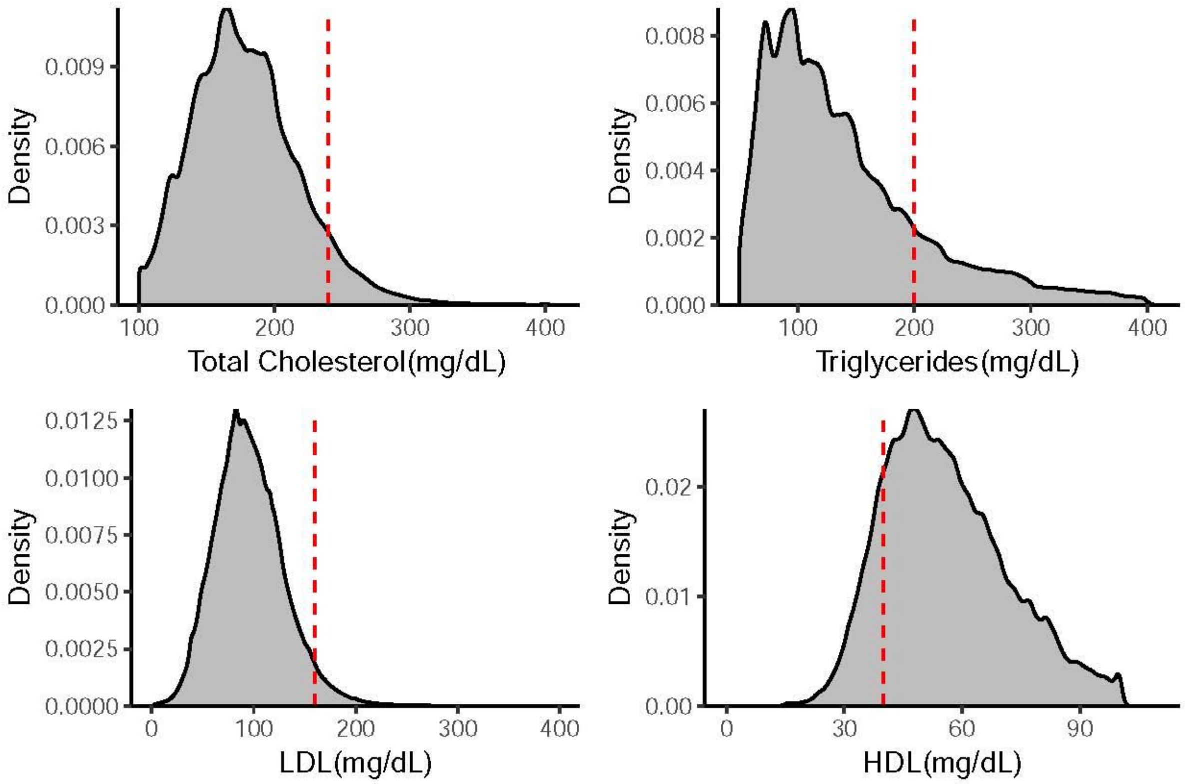

© 2021 Lu Y et al. *JAMA Network Open.*

eFigure 3. Availability of Lipid-Lowering Medications (Statin, Non-Statin, Traditional Chinese Medicine) Among 3,041 Primary Care Institutions, by Type of Site and Economic Region

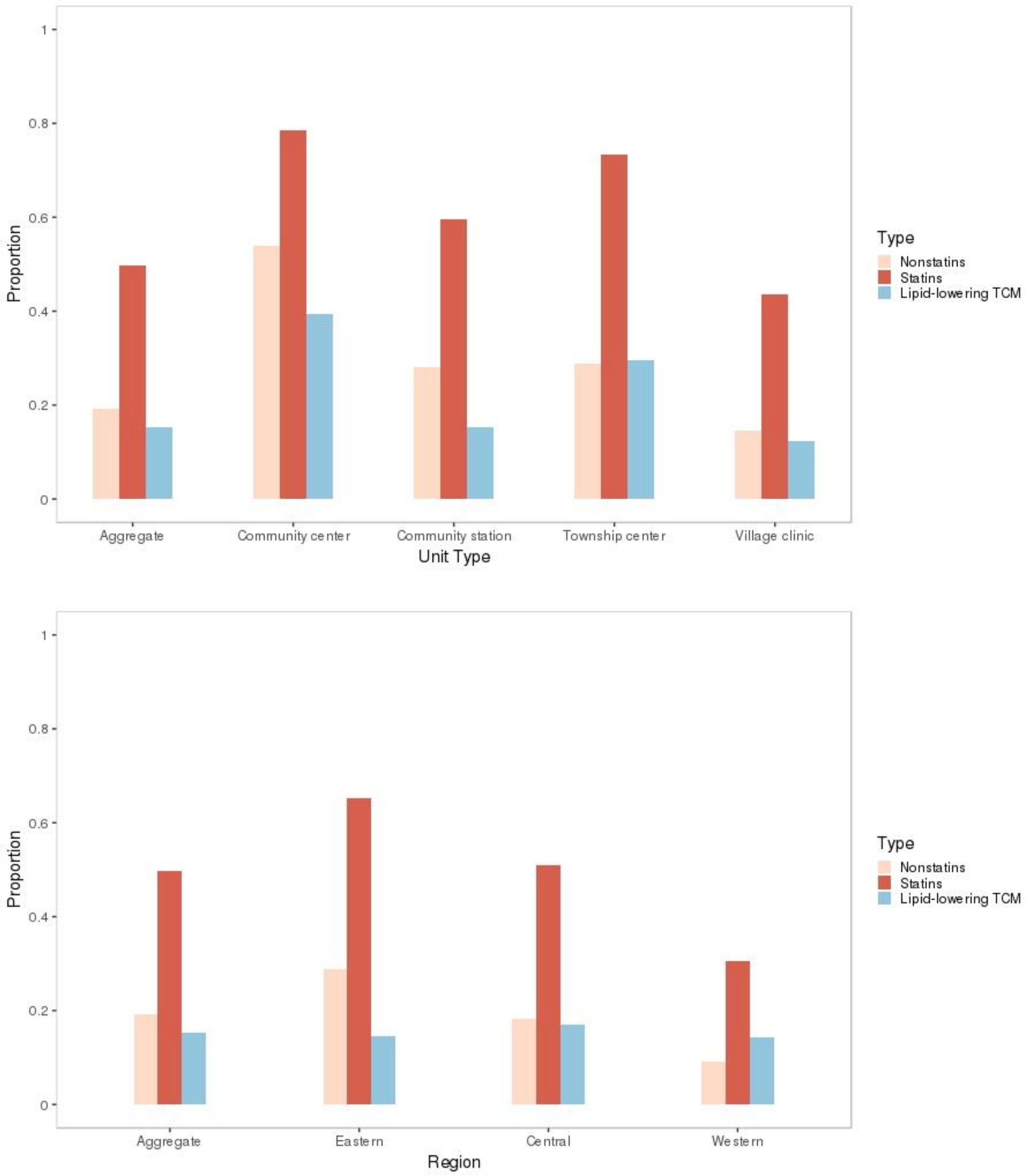

© 2021 Lu Y et al. *JAMA Network Open.*
