# Supplementary material for: Prevalence of Dyslipidemia and Availability of Lipid-Lowering Medications Among Primary Health Care Settings in China
Source: JAMA Netw Open. 2021 Sep 29;4(9):e2127573. doi: 10.1001/jamanetworkopen.2021.27573 (PMC8482054; doi:10.1001/jamanetworkopen.2021.27573)
Supplement: Supplement 2. — China Patient-Centered Evaluative Assessment of Cardiac Events Million Persons Project (China-PEACE MPP) Collaborative Group Members [file jamanetwopen-e2127573-s002.pdf]

Supplemental Online Content: Nonauthor Collaborators

*Indicates required information. Only first name, last name, and suffix will appear in PubMed.

*Group Name: China Patient-Centered Evaluative Assessment of Cardiac Events Million Persons Project (China-PEACE MPP) Collaborative Group							
***First Name and Middle Initial(s)**	***Last Name**	***Suffix (eg, Jr, III)**	Academic Degrees	Institution	Location (city, state/province, country)	Role or Contribution, eg, chair, principal investigator	Group (if more than 1 Group listed in the byline) and/or Subgroup (eg, Steering Committee)
Chun	Huang			Beijing Center for Diseases Prevention and Control			
Zhong	Dong			Beijing Center for Diseases Prevention and Control			
Bo	Jiang			Beijing Center for Diseases Prevention and Control			
Zhigang	Guo			Tianjin Chest Hospital			
Yingyi	Zhang			Tianjin Chest Hospital			
Jixin	Sun			Hebei Center for Diseases Prevention and Control			
Yuhuan	Liu			Hebei Center for Diseases Prevention and Control			
Zeping	Ren			Shanxi Center for Diseases Prevention and Control			
Yaqing	Meng			Shanxi Center for Diseases Prevention and Control			
Zhifen	Wang			Inner Mongolia Center for Diseases Prevention and Control			
Yunfeng	Xi			Inner Mongolia Center for Diseases Prevention and Control			
Liying	Xing			Liaoning Center for Diseases Prevention and Control			
Yuanmeng	Tian			Liaoning Center for Diseases Prevention and Control			
Jianwei	Liu			Jilin Center for Diseases Prevention and Control			
Yao	Fu			Jilin Center for Diseases Prevention and Control			

Supplemental Online Content: Nonauthor Collaborators

*Indicates required information. Only first name, last name, and suffix will appear in PubMed.

*First Name and Middle Initial(s)	*Last Name	*Suffix (eg, Jr, III)	Academic Degrees	Institution	Location (city, state/province, country)	Role or Contribution, eg, chair, principal investigator	Group (if more than 1 Group listed in the byline) and/or Subgroup (eg, Steering Committee)
Ting	Liu			Jilin Center for Diseases Prevention and Control			
Wei	Sun			Heilongjiang Center for Diseases Prevention and Control			
Shichun	Yan			Heilongjiang Center for Diseases Prevention and Control			
Lin	Jin			Heilongjiang Center for Diseases Prevention and Control			
Yang	Zheng			Shanghai Center for Diseases Prevention and Control			
Jing	Wang			Shanghai Center for Diseases Prevention and Control			
				Jiangsu Center for Diseases Prevention and Control			
Jing	Yan			Zhejiang Provincial People's Hospital			
Xiaoling	Xu			Zhejiang Provincial People's Hospital			
Yeji	Chen			Anhui Center for Diseases Prevention and Control			
Xiuya	Xing			Anhui Center for Diseases Prevention and Control			
Luan	Zhang			Anhui Center for Diseases Prevention and Control			
Wenling	Zhong			Fujian Center for Diseases Prevention and Control			
Xin	Fang			Fujian Center for Diseases Prevention and Control			
Liping	Zhu			Jiangxi Center for Diseases Prevention and Control			

Supplemental Online Content: Nonauthor Collaborators

*Indicates required information. Only first name, last name, and suffix will appear in PubMed.

*First Name and Middle Initial(s)	*Last Name	*Suffix (eg, Jr, III)	Academic Degrees	Institution	Location (city, state/province, country)	Role or Contribution, eg, chair, principal investigator	Group (if more than 1 Group listed in the byline) and/or Subgroup (eg, Steering Committee)
Yan	Xu			Jiangxi Center for Diseases Prevention and Control			
Xiaolei	Guo			Shandong Center for Diseases Prevention and Control			
Chunxiao	Xu			Shandong Center for Diseases Prevention and Control			
Gang	Zhou			Henan Center for Diseases Prevention and Control			
Lei	Fan			Henan Center for Diseases Prevention and Control			
Minjie	Qi			Henan Center for Diseases Prevention and Control			
Shuzhen	Zhu			Hubei Center for Diseases Prevention and Control			
Junfeng	Qi			Hubei Center for Diseases Prevention and Control			
Junlin	Li			Hubei Center for Diseases Prevention and Control			
Li	Yin			Hunan Center for Diseases Prevention and Control			
Qiong	Liu			Hunan Center for Diseases Prevention and Control			
Qingshan	Geng			Guangdong Provincial People's Hospital			
Yingqing	Feng			Guangdong Provincial People's Hospital			
Jiabin	Wang			Guangdong Provincial People's Hospital			
Hong	Wen			The First Affiliated Hospital of Guangxi Medical University			
Xuemei	Han			Health Commission of Hainan			

Supplemental Online Content: Nonauthor Collaborators

*Indicates required information. Only first name, last name, and suffix will appear in PubMed.

***First Name and Middle Initial(s)**	***Last Name**	***Suffix (eg, Jr, III)**	Academic Degrees	Institution	Location (city, state/province, country)	Role or Contribution, eg, chair, principal investigator	Group (if more than 1 Group listed in the byline) and/or Subgroup (eg, Steering Committee)
Puyu	Liu			Hainan Center for Diseases Prevention and Control			
Xianbin	Ding			Chongqing Center for Diseases Prevention and Control			
Jie	Xu			Chongqing Center for Diseases Prevention and Control			
Ying	Deng			Sichuan Center for Diseases Prevention and Control			
Jun	He			Sichuan Center for Diseases Prevention and Control			
Gui'e	Liu			Guizhou Provincial People's Hospital			
Chenxi	Jiang			Guizhou Provincial People's Hospital			
Shun	Zha			Yunnan Center for Diseases Prevention and Control			
Cangjiang	Yang			Yunnan Center for Diseases Prevention and Control			
Guoxia	Bai			Tibet Center for Diseases Prevention and Control			
Yue	Yu			Tibet Center for Diseases Prevention and Control			
Zongji	Tashi			Tibet Center for Diseases Prevention and Control			
Lin	Qiu			Shanxi Center for Diseases Prevention and Control			
Zhiping	Hu			Shanxi Center for Diseases Prevention and Control			
Hupeng	He			Gansu Center for Diseases Prevention and Control			

Supplemental Online Content: Nonauthor Collaborators

*Indicates required information. Only first name, last name, and suffix will appear in PubMed.

***First Name and Middle Initial(s)**	***Last Name**	***Suffix (eg, Jr, III)**	Academic Degrees	Institution	Location (city, state/province, country)	Role or Contribution, eg, chair, principal investigator	Group (if more than 1 Group listed in the byline) and/or Subgroup (eg, Steering Committee)
Jing	Zhang			Gansu Center for Diseases Prevention and Control			
Minru	Zhou			Qinghai Center for Diseases Prevention and Control			
Xiaoping	Li			Qinghai Center for Diseases Prevention and Control			
Jianhua	Zhao			Ningxia Center for Diseases Prevention and Control			
Shaoning	Ma			Ningxia Center for Diseases Prevention and Control			
Yitong	Ma			The First Affiliated Hospital of Xinjiang Medical University			
Ying	Huang			The First Affiliated Hospital of Xinjiang Medical University			
Yuchen	Zhang			The First Affiliated Hospital of Xinjiang Medical University			
Fanka	Li			Xinjiang Corps Center for Diseases Prevention and Control			
Jiacong	Shen			Xinjiang Corps Center for Diseases Prevention and Control			
